# Mitofusin-mediated ER stress triggers neurodegeneration in *pink1*/*parkin* models of Parkinson's disease

**DOI:** 10.1038/cddis.2016.173

**Published:** 2016-06-23

**Authors:** I Celardo, A C Costa, S Lehmann, C Jones, N Wood, N E Mencacci, G R Mallucci, S H Y Loh, L M Martins

**Affiliations:** 1 MRC Toxicology Unit, Lancaster Road, Leicester LE1 9HN, UK; 2 Department of Molecular Neuroscience, Institute of Neurology, University College London, London WC1N 3BG, UK; 3 Department of Clinical Neurosciences, University of Cambridge, Clifford Allbutt Building, Cambridge Biomedical Campus, Cambridge CB1 0HN, UK

## Abstract

Mutations in *PINK1* and *PARKIN* cause early-onset Parkinson's disease (PD), thought to be due to mitochondrial toxicity. Here, we show that in *Drosophila pink1* and *parkin* mutants, defective mitochondria also give rise to endoplasmic reticulum (ER) stress signalling, specifically to the activation of the protein kinase R-like endoplasmic reticulum kinase (PERK) branch of the unfolded protein response (UPR). We show that enhanced ER stress signalling in *pink1* and *parkin* mutants is mediated by mitofusin bridges, which occur between defective mitochondria and the ER. Reducing mitofusin contacts with the ER is neuroprotective, through suppression of PERK signalling, while mitochondrial dysfunction remains unchanged. Further, both genetic inhibition of *dPerk*-dependent ER stress signalling and pharmacological inhibition using the PERK inhibitor GSK2606414 were neuroprotective in both *pink1* and *parkin* mutants. We conclude that activation of ER stress by defective mitochondria is neurotoxic in *pink1* and *parkin* flies and that the reduction of this signalling is neuroprotective, independently of defective mitochondria. A video abstract for this article is available online in the supplementary information

Recently, endoplasmic reticulum (ER) stress, and in particular dysregulation of the protein kinase R-like endoplasmic reticulum kinase (PERK) branch of the unfolded protein response (UPR) have emerged as major toxic processes in protein misfolding neurodegenerative disorders (reviewed in Halliday and Mallucci^1^). Overactivation of PERK signalling is a feature of post-mortem brains of patients with Alzheimer's and Parkinson's diseases and the tauopathies, frontotemporal dementia (FTD) and Progressive Supranuclear Palsy (reviewed in Scheper and Hoozemans^2^). In mice with prion disease^3^ and FTD-like pathology,^4^ sustained activation of the PERK branch of the UPR leads to chronic reduction in global protein synthesis rates in the brain. The reduction in translation of vital proteins leads to neuronal death, which is rescued by inhibition of the pathway at the level of PERK^3, 4, 5^ or downstream effectors.^6^ In Parkinson's disease (PD), mitochondrial dysfunction, due to loss of function of PTEN-induced putative kinase 1 (PINK1) or PARKIN, is a central pathogenic process (reviewed in Celardo *et al.*^7^). Mitochondrial impairment has been described to trigger ER stress,^8^ but whether and how these two processes converge and/or contribute to neurodegeneration remains unknown. *Drosophila pink1* or *parkin* mutants show neurodegeneration, a crushed thorax phenotype and mitochondrial dysfunction.^9, 10^

We therefore asked: first, whether ER stress occurs in *Drosophila pink1*/*parkin* models of PD and contributes to the neurodegenerative phenotype, and second: to what extent, if any, ER stress is driven by defective mitochondria? We found that mitochondrial dysfunction in *pink1* or *parkin* mutant flies does activate the PERK branch of the UPR through the formation of mitofusin bridges between defective mitochondria and the ER. Further, we found that inhibiting PERK signalling genetically and pharmacologically, or through the reduction of mitofusin bridges was neuroprotective in *pink1* and *parkin* mutant flies, irrespective of the persistence of defective mitochondria.

## Results

### *pink1* and *parkin* mutants show activation of the PERK branch of the UPR

**Figure 1:**
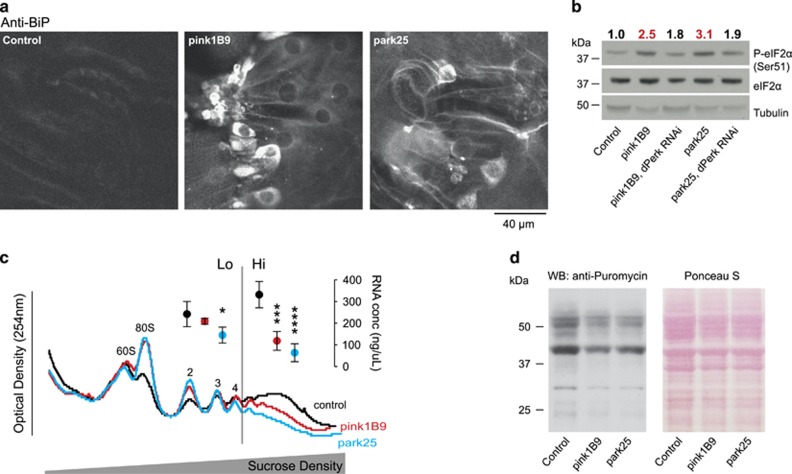
Activation of phospho-eIF2*α* signalling and attenuation of translation in *pink1* and *parkin* mutant flies. (**a**) Increased levels of BiP in the body wall muscle of *pink1* and *parkin* mutant larvae. Representative confocal images with the indicated genotype stained with *α*-BiP antibody are shown. (**b**) Increased levels of phospho-eIF2*α* in *pink1* and *parkin* mutant flies are reduced by knockdown of *dPerk*. Whole-fly lysates were analysed using the indicated antibodies. Ratios of signal intensity between phospho and total-eIF2*α* are shown at the top. (**c**) Polysomal distribution of mRNAs of young adult male flies showing individual ribosomal subunits and the polysome peaks. RNA concentrations were measured from the low (Lo) and high (Hi) translation fractions (mean±S.D., asterisks, one-way ANOVA with Dunnett's multiple comparison test; *n*=4). (**d**) Reduced puromycin incorporation in nascent proteins in *pink1* and *parkin* mutant flies. Whole-fly lysates were analysed with an anti-puromycin antibody and equivalent protein loading was assessed by Ponceau S staining of the membranes. Genotypes for (**b**) Control: *daGAL4*; pink1^B9^: *pink1*^*B9*^*,daGAL4*; park^25^: *park*^*25*^*/park*^*25*^*,daGAL4*. RNAi *dPerk* was driven by *da*GAL4. (**a**, **c** and **d**) Control: *w*^*1118*^

We first examined *pink1* and *parkin* mutants for evidence of ER stress and UPR activation. We found increased levels of chaperone-binding immunoglobulin protein (BiP), a marker for ER stress activation, in the body wall muscle cells^11^ of both *pink1* and *parkin* mutant larvae compared with wild-type controls (Figure 1a). Upon ER stress, BiP dissociates from PERK, which dimerizes and autophosphorylates. Phospho-PERK in turn phosphorylates eukaryotic initiation factor 2 alpha (eIF2*α*), which leads to inhibition of global protein translation rates at the level of initiation. We found increased levels of phospho-eIF2*α* in *pink1* and *parkin* mutants, which were reduced upon knockdown of *dPerk* (Figure 1b), consistent with its activation through PERK signalling and raised levels of BiP.

The relative translation rate of an mRNA can be deduced from the number of ribosomes (polysomes) it recruits. We found an overall reduction of the number of polysomes bound to mRNAs in adult *pink1* and *parkin* mutants by polysomal profiling (Figure 1c), consistent with a decrease in global translation rates. Additionally, we detected a decrease in protein synthesis, measured by assessing the incorporation of puromycin, a Tyr-tRNA mimetic, into newly translated proteins (Figure 1d).^12^ These findings support activation of signalling through the PERK branch of ER stress in *pink1* and *parkin* mutant flies.

### *pink1* and *parkin* mutants show an enhanced association between defective mitochondria and the ER

**Figure 2:**
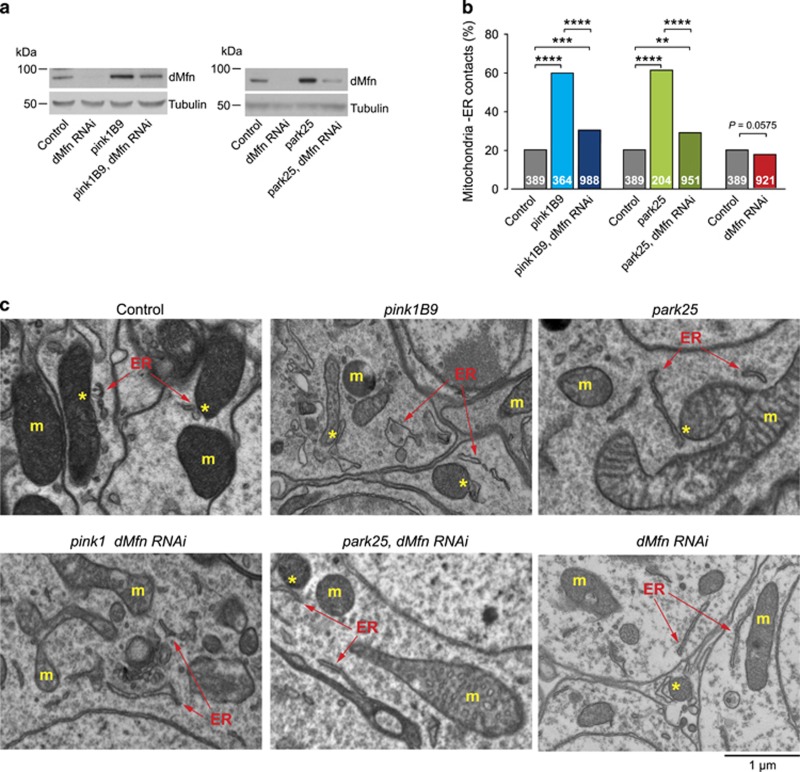
dMfn mediates the recruitment of defective mitochondria to ER contact points in *pink1* or *parkin* mutants. (**a**) The RNAi-mediated suppression of *dMfn* reduces dMfn protein levels in *pink1* and *parkin* mutants. Whole-fly lysates were analysed using the indicated antibodies. (**b** and **c**) Quantification of mitochondria–ER contacts in adult *Drosophila* brains (asterisks, chi-square two-tailed, 95% confidence intervals) (**b**), and representative electron microscopy images (**c**). Yellow asterisks show mitochondria in contact with ER (arrows). ER, endoplasmic reticulum; m, mitochondria. Genotypes for (**a**) Control: *daGAL4*; pink1^B9^: *pink1*^*B9*^*,daGAL4*; park^25^: *park*^*25*^*/park*^*25*^*,daGAL4*. RNAi *dMfn* was driven by *da*GAL4. (**b** and **c**) Control: *elavGAL4*; pink1^B9^: *pink1*^*B9*^*,elavGAL4*; park^25^: *park*^*25*^*/park*^*25*^*,elavGAL4*. RNAi *dMfn* was driven by *elav*GAL4

**Figure 3:**
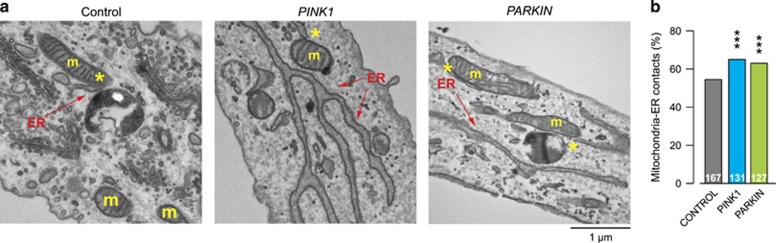
Increase in mitochondria–ER contact points in *PINK1* or *PARKIN* mutant fibroblasts. (**a** and **b**) Quantification of mitochondria–ER contacts in human fibroblasts (asterisks, chi-square two-tailed, 95% confidence intervals) (**b**), and representative electron microscopy images (**a**). Yellow asterisks show mitochondria in contact with ER (arrows). ER, endoplasmic reticulum; m, mitochondria. *PINK1*: c.261_276del16; p.T90LfsX12 (homozygous); *PARKIN*: deletion of exons 3 and 4 (homozygous)

We next asked whether there is cross-talk between dysfunctional mitochondria and activation of PERK signalling. Pink1 and Parkin mediate the ubiquitination and degradation of the profusion factor mitofusin (dMfn) on the outer surface of mitochondria; and *pink1* or *parkin* mutant flies show an accumulation of dMfn.^13^ Mitofusin modulates mitochondrial fusion and the tethering of these organelles to the ER.^14^ To test whether the accumulation of dMfn in both *pink1* and *parkin* mutants affected the proximity between mitochondria and the ER, we quantified mitochondria–ER contacts using a previously described assay.^15^ We first confirmed the previously reported accumulation of dMfn in *pink1* and *parkin* mutant flies, which could be partially reversed upon *dMfn* RNA interference (RNAi) (Figure 2a). Ultrastructural analysis of fly brains revealed that both *pink1* and *parkin* mutants show significant increases in mitochondria–ER contact sites that can be suppressed upon *dMfn* knockdown (Figures 2b and c). Further, analysis of mitochondria–ER contacts in cultured human fibroblasts obtained from PD patients carrying homozygous *PINK1* or *PARKIN* pathogenic mutations also confirmed that mutations in *PINK1* or *PARKIN* are associated with an increased level of contacts between these two organelles (Figures 3a and b).

### Reducing the association between defective mitochondria and the ER attenuates PERK/eIF2*α* signalling and is neuroprotective in *pink1* and *parkin* mutants

**Figure 4:**
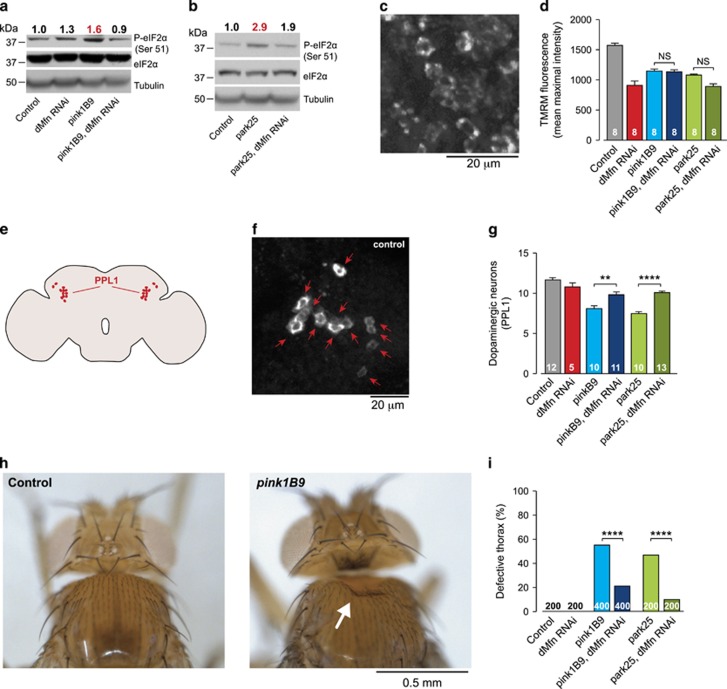
Suppressing *dMfn* in *pink1* or *parkin* mutants attenuates eIF2*α* signalling and blocks neurodegeneration. (**a** and **b**) Decreased levels of phospho-eIF2*α* upon RNAi-mediated suppression of *dMfn* in *pink1* (**a**) and *parkin* (**b**) mutant flies. Whole-fly lysates were analysed using the indicated antibodies. Ratios of signal intensity between phospho and total-eIF2*α* are shown at the top. (**c** and **d**) RNAi-mediated suppression of *dMfn* does not prevent the loss of Δψm in *pink1* or *parkin* mutants. Representative confocal image of a whole mounted control brain showing neurons loaded with TMRM (**c**). Quantification of Δψm in the brains of the indicated genotypes (**d**) (mean±S.E.M.; asterisks, one-way ANOVA with Bonferroni's multiple comparison test). (**e**–**g**) The RNAi-mediated suppression of *dMfn* rescues the loss of dopaminergic neurons in the PPL1 cluster of *pink1* and *parkin* mutant flies. Schematic diagram of a fly brain in sagittal orientation indicating the PPL1 cluster of dopaminergic neurons in red (**e**). Anti-TH staining showing cell bodies of PPL1 neurons in a representative control brain (**f**) and quantification of the PPL1 cluster neurons (**g**) (mean±S.E.M.; asterisks, one-way ANOVA with Bonferroni's multiple comparison test). (**h** and **i**) Suppression of the thoracic defects of *pink1* and *parkin* mutants by RNAi-mediated suppression of *dMfn.* Representative images of normal and defective thorax in *pink1* mutants, the arrow points to a thoracic defect (**h**). Quantification of the thoracic defects (**i**) in the indicated genotypes (asterisks, chi-square two-tailed, 95% confidence intervals). Genotypes for (**a**, **b**, **i**) Control: *daGAL4*; pink1^B9^: *pink1*^*B9*^*,daGAL4*; park^25^: *park*^*25*^*/park*^*25*^*,daGAL4*. RNAi *dMfn* was driven by *da*GAL4. (**c, d, f, g**) Control: *elavGAL4*; pink1^B9^: *pink1*^*B9*^*,elavGAL4*; park^25^: *park*^*25*^*/park*^*25*^*,elavGAL4*. RNAi *dMfn* was driven by *elav*GAL4. (**h**) Control: *w*^*1118*^

Next, we tested whether the association of defective mitochondria with the ER upon the loss of *pink1* or *parkin* affects the PERK-dependent ER stress pathway. Decreasing mitochondria–ER contacts by *dMfn* knockdown reduced levels of phospho-eIF2*α* in *pink1* and *parkin* mutants (Figures 4a and b), indicating that it is the association of defective mitochondria with the ER that activates ER stress in these flies.

It has been previously shown that the knockdown of *dMfn* is able to rescue the mitochondrial morphology phenotypes of *pink1* and *parkin* mutant flies.^16, 17^ We found that while reducing mitofusin reduced ER stress, this knockdown did not rescue defective mitochondrial function. Thus, loss of mitochondrial membrane potential (Δψm) (Figures 4c and d) as seen in *pink1* and *parkin* mutants^18^ is not reversed by the knockdown of *dMfn* in neurons (Figure 4d). Despite this, *dMfn* knockdown is sufficient to suppress both the loss of protocerebral posterior lateral 1 (PPL1) cluster of dopaminergic neurons (Figures 4e, f and g) and the crushed-thorax phenotypes (Figures 4h and i) of *pink1* and *parkin* mutants. Taken together, these results support that neurodegeneration associated with *pink1* and *parkin* can be prevented by *dMfn* knockdown, in the absence of mitochondrial functional improvement.

### Inhibiting activation of PERK/eIF2*α* signalling in *pink1* and *parkin* mutants prevents neurodegeneration

**Figure 5:**
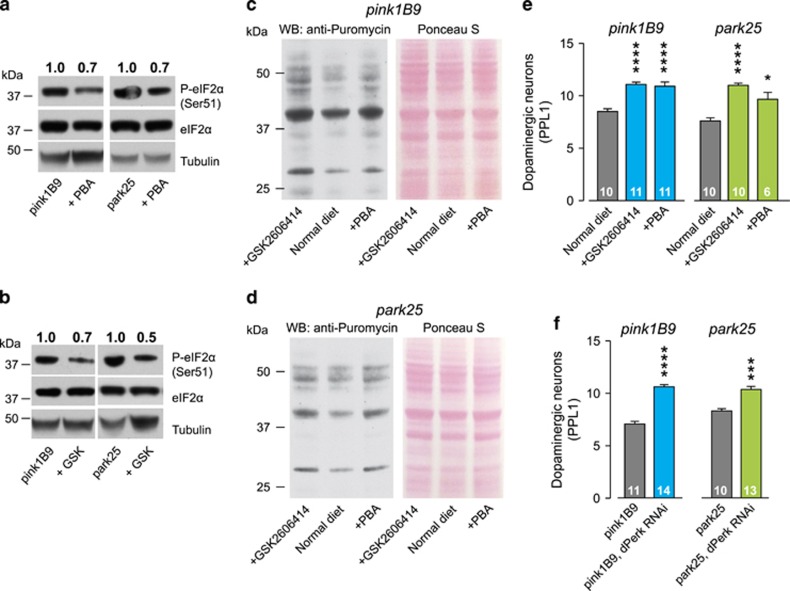
Inhibiting PERK/eIF2*α* signalling in *pink1* and *parkin* mutants prevents neurodegeneration. (**a** and **b**) The chemical chaperone PBA (**a**) and the PERK inhibitor GSK2606414 (**b**) decrease the levels of phospho-eIF2*α* in *pink1* and *parkin* mutant flies. Whole-fly lysates were analysed using the indicated antibodies. Ratios of signal intensity between phospho and total-eIF2*α* are shown at the top. (**c** and **d**) Recovery of puromycin incorporation in nascent proteins in *pink1* (**c**) and *parkin* (**d**) mutant flies upon dietary supplementation with PBA or GSK2606414. Whole-fly lysates were analysed with an anti-puromycin antibody and equivalent protein loading was assessed by Ponceau S staining of the membranes. (**e**) The chemical chaperone PBA and the PERK inhibitor GSK2606414 rescue the loss of dopaminergic neurons in the PPL1 cluster of *pink1* and *parkin* mutant flies (mean±S.E.M.; asterisks, one-way ANOVA with Bonferroni's multiple comparison test). (**f**) The RNAi-mediated suppression of *dPerk* rescues the loss of dopaminergic neurons in the PPL1 cluster of *pink1* and *parkin* mutant flies (mean±S.E.M.; asterisks, one-way ANOVA with Bonferroni's multiple comparison test). Genotypes for (**f**) pink1^B9^: *pink1*^*B9*^*,elavGAL4*; park^25^: *park*^*25*^*/park*^*25*^*,elavGAL4*. RNAi *dPerk* was driven by *elav*GAL4

*Drosophila pink1* and *parkin* mutants show a loss of the PPL1 cluster of dopaminergic neurons.^9, 10^ To determine whether the dopaminergic neuronal loss in these mutants is caused by the activation of ER stress, we used both pharmacological and genetic tools to block its activation. The diet of *pink1* and *parkin* mutants was supplemented with 4-phenylbutyric acid (PBA), a chemical chaperone that attenuates ER stress^19, 20^ or the selective PERK inhibitor compound, GSK2606414.^21^ Both compounds reduced the levels of phospho-eIF2*α* in *pink1* and *parkin* mutants (Figures 5a and b), increased puromycin incorporation in *pink1* (Figure 5c) and *parkin* (Figure 5d) mutants, and were neuroprotective, preventing PPL1 neuronal loss (Figure 5e). Further, genetic knockdown of *dPerk* was similarly neuroprotective (Figure 5f). The data support that ER stress activation, through PERK signalling, contributes to PD-associated neurodegenerative phenotypes in *pink1* and *parkin* mutants, consistent with data in other models of neurodegeneration including prion disease^3, 5^ or tauopathy mice^4^ and a *Drosophila* model of Amyotrophic Lateral Sclerosis.^22^ Further, they support pharmacological inhibition of PERK signalling as neuroprotective.

## Discussion

Our results show that the loss of *pink1* or *parkin* leads to the activation of ER stress through a direct interaction between mitochondria and the ER, promoted by increased levels of dMfn. We observed an increase in contacts between mitochondria and the ER in both flies and cultured human fibroblasts from PD patients with *PINK1* or *PARKIN* mutations. However, further analysis of these fibroblasts did not detect any alterations in mitochondrial function or ER stress signalling (data not shown). It is possible that defective mitochondria at the ER contact points are causing the activation of ER stress; therefore, the long-term adaptation to cell-culture conditions could explain why these fibroblasts do not display mitochondrial dysfunction and therefore fail to activate ER stress. As mitochondrial function is compromised in both *pink1* and *parkin* mutant flies, it is conceivable that the activation of ER stress in these mutants is linked to the functional status of mitochondria at ER contacts. Further, mitofusins can tether two mitochondria together as well as tether mitochondria to the ER (reviewed in Rowland and Voeltz^23^). They therefore have complex roles in both intra- and inter-organelle coupling. Indeed, the ablation of Mitofusin 2 in mouse anorexigenic proopiomelanocortic neurons reduces the contacts between functional mitochondria and the ER leading to ER stress.^15^ In contrast, we show that the loss of contacts between dysfunctional mitochondria and the ER in *pink1* or *parkin* mutants with downregulated *dMfn* ameliorates the PERK branch of ER stress signalling. As these mutants show mitochondrial dysfunction, we propose that it is not only the quantity, but also the quality, of mitochondria at ER contact points that modulates ER stress and may underlie this discrepancy.

In conclusion, we show how two major processes, mitochondrial dysfunction and ER stress converge in mediating neurodegeneration in models of PD, mediated by inter-organelle interactions. Further, we show that modulating PERK signalling may represent a valid strategy for pathologies where mitochondrial impairment and the resulting ER stress is also a major pathogenic mechanism in neuronal demise, as in Parkinson's and related diseases.

## Materials and Methods

### Genetics and *Drosophila* strains

Fly stocks and crosses were maintained on standard cornmeal agar media at 25 °C. The strains used were *pink1*^*B9*^, *park*^*25*^, *daGAL4* (kind gifts from A Whitworth, MRC, Centre for Developmental and Biomedical Genetics, University of Sheffield, Sheffield, UK), *w*^*1118*^, *elavGAL4* (Bloomington Stock Centre), and RNAi lines: *dMfn* (ID: 105261) and *dPerk* (ID: 16427) (Vienna *Drosophila* RNAi Centre). All the experiments on adult flies were performed with males.

### Protein translation assays

For polysome profiling, fifty 3-day-old flies were homogenized on ice in 600 *μ*l of cold solubilization buffer (300 mM NaCl, 15 mM MgCl_2_, 15 mM Tris-HCl pH 7.5, 2 mM DTT, 0.1 mg/ml cycloheximide, 1% Triton X-100, 25 *μ*l/ml Super-ase-In) and the homogenates were centrifuged at 21 000 *g* for 10 min. The supernatants were recovered and applied to the top of a 10–50% sucrose gradient in high salt resolving buffer (300 mM NaCl, 15 mM MgCl_2_, 15 mM Tris-HCl pH 7.5, 2 mM DTT, 0.1 mg/ml cycloheximide). Ribosomal subunits and polysomes were separated by centrifugation at 25 0000 *g* for 2 h at 4 °C. Gradients were fractionated using a Teledyne Isco density gradient fractionator, Foxy R1 (Teledyne Isco, NE, USA) with constant measurement of absorbance at 254 nm. Ten fractions were isolated per run, and RNA was prepared from each one of those fractions using guanidine hydrochloride with ethanol precipitation. To measure nascent protein synthesis, five 3-day-old flies were homogenized in cold solubilization buffer without cycloheximide, supplemented with 100 *μ*M puromycin. Lysates were incubated at 4 °C for 5 min and mixed with 4 × LDS loading buffer.

### Cell culture

Human primary fibroblasts were cultured in DMEM (Gibco BRL, Waltham, MA, USA); media was supplemented with 10% heat-inactivated FCS (Invitrogen, Paisley, UK), 100 U/ml penicillin (Gibco BRL), 100 *μ*g/ml streptomycin (Gibco BRL) and 50 *μ*g/ml gentamicin (Sigma, Gillingham, UK). The cells were maintained at 37 °C in 5% CO_2_ in culture medium.

### Ethics statement

All human primary fibroblast cells were generated *in vitro* after written informed consent using protocols approved by the ULCH research and development department and the City Road and Hampstead ethics committee, London, UK.

### Protein extraction and western blotting

Protein extracts from whole flies were prepared by grinding flies in lysis buffer (100 mM KCl, 20 mM Hepes at pH 7.5, 5% (v/v) glycerol, 10 mM EDTA, 0.1% (v/v) Triton X-100, 10 mM DTT, 1 *μ*g/ml leupeptin, 1 *μ*g/ml antipain, 1 *μ*g/ml chymostatin and 1 *μ*g/ml pepstatin). Protein extracts from whole flies for phospho and total-eIF2*α* immunoblots were prepared by grinding flies in lysis buffer (20 mM Tris at pH 7.5, 150 mM NaCl, 1% (v/v) Nonidet-40, 0.5% (w/v) Sodium deoxycholate, 0.1% (w/v) SDS) containing phosphatase inhibitor cocktail tablets PhosSTOP (Roche, Mannheim, Germany) and the protease inhibitors leupeptin, antipain, chymostatin and pepstatin (Sigma) at the manufacturer's recommended dilution. The suspensions were cleared by centrifugation at 21 000 *g* for 10 min at 4 °C and protein concentrations of the supernatants were measured using the Bradford assay (Bio-Rad, Hemel Hempstead, UK). All supernatants were mixed with 4 × LDS loading buffer. For SDS-PAGE, equivalent amounts of proteins were resolved on 4–12% or 10% Precast Gels (Invitrogen) and transferred onto PVDF membranes (Millipore, Billerica, MA, USA). The membranes were blocked in TBS-T (0.15 M NaCl, 10 mM Tris-HCl pH 7.5, 0.1% Tween-20) containing 10% (w/v) dried non-fat milk for 1 h at room temperature, probed with the indicated primary antibody before being incubated with the appropriate HRP-conjugated secondary antibody. Antibody complexes were visualized by Pierce enhanced chemiluminescence (ECL). The levels of phospho-eIF2*α* were calculated as a ratio to the total-eIF2*α* levels, using the ImageJ software (http://imagej.nih.gov/ij/; provided in the public domain by the National Institutes of Health, Bethesda, MD, USA).

### Antibodies

Primary antibodies employed in this study were EIF2S1 (ser51) (Abcam, Cambridge, UK, ab32157), EIF2S1 (Abcam, ab26197), *α*-tubulin (Sigma, T6074), GAPDH (Sigma, G8795), BiP (BT-GB-143S, Babraham Bioscience Technologies, Cambridge, UK), TH (22941, Immunostar, Hudson, WI, USA), Puromycin (Millipore, MABE343), dMfn (a gift from A. Whitworth, MRC, Centre for Developmental and Biomedical Genetics, University of Sheffield).

### Confocal microscopy

For anti-Bip immunostaining, third-instar larvae were dissected, fixed in 4% paraformaldehyde, stained with anti-BiP (1 : 50) essentially as previously described.^11^

### Microscopy-based assessment of mitochondrial function

Measurements of Δψm in fly brains were performed using tetramethylrhodamine (TMRM) as previously described.^18^ Briefly, fly brains were loaded for 40 min at room temperature with 40 nM TMRM in loading buffer (10 mM HEPES pH 7.35, 156 mM NaCl, 3 mM KCl, 2 mM MgSO_4_, 1.25 mM KH_2_PO_4_, 2 mM CaCl_2_, 10 mM glucose) and the dye was present during the experiment. In these experiments, TMRM is used in the redistribution mode to assess Δψm; and therefore, a reduction in TMRM fluorescence represents mitochondrial depolarization. Confocal images were obtained using a Zeiss 510 confocal microscope (ZEISS, Jena, Germany) equipped with a × 40 oil immersion objective. Illumination intensity was kept to a minimum (at 0.1–0.2% of laser output) to avoid phototoxicity; and the pinhole was set to give an optical slice of 2 *μ*m. Fluorescence was quantified by exciting TMRM using the 565-nm laser and measured above 580 nm. Z-stacks of five fields of 300 *μ*m^2^ each per brain were acquired, and the mean maximal fluorescence intensity was measured for each group.

### Electron microscopy

For transmission electron microscopy (TEM), adult fly brains and human fibroblast were fixed overnight in 0.1 M sodium cacodylate buffer (pH 7.4) containing 2% paraformaldehyde, 2.5% glutaraldehyde and 0.1% Tween-20. Samples were post-fixed for 1 h at room temperature in a solution containing 1% osmium tetroxide and 1% potassium ferrocyanide. After fixation, samples were stained *en bloc* with 5% aqueous uranyl acetate overnight at room temperature; the samples were then dehydrated via a series of ethanol washes and embedded in TAAB epoxy resin (TAAB Laboratories Equipment Ltd., Aldermaston, UK). Semi-thin sections were stained with toluidine blue, and areas of the sections were selected for ultramicrotomy. Ultrathin sections were stained with lead citrate and imaged using a MegaView 3 digital camera and iTEM software (Olympus Soft Imaging Solutions GmbH, Münster, Germany) in a Jeol 100-CXII electron microscope (Jeol UK Ltd., Welwyn Garden City, UK).

### Defective thorax analysis

Visual assessment of thoracic indentations (defective thorax) was assessed essentially as a binary assay: first, we ask if a fly has a defective thorax or not; second, we use chi-square statistics to determine whether the degree (percentage) of crushed thorax in the populations under analysis is significantly different.

### Drug treatments

Drugs used were incorporated into the fly food. Flies treated with PBA (Merck Millipore, MA, USA) were raised in PBA-containing food at a concentration of 7.5 mM. Flies treated with GSK2606414 (Merck Millipore) were transferred to drug-containing food (10 *μ*M) up to 24 h after hatching. The adult flies were kept in drug-containing food throughout lifespan.

### Analysis of dopaminergic neurons

Fly brains were dissected from 20-day-old flies and stained for anti-tyrosine hydroxylase (TH, Immunostar) as previously described.^10^ Brains were positioned in PBS+0.1% Triton in a coverslip clamp chamber (ALA Scientific Instruments Inc., Farmingdale, NY, USA) using a harp made of platinum wire and nylon string and imaged by confocal microscopy. Tyrosine hydroxylase-positive PPL1 cluster neurons were counted per brain hemisphere. Data acquired for the assessment of each genotype were obtained as a single experimental set before statistical analysis.

### Statistical analyses

Descriptive and inferential statistical analyses were performed using GraphPad Prism 5 (www.graphpad.com). Data are presented as the mean values, and the error bars indicate±S.D. or±S.E.M. as indicated. The number of biological replicates per experimental variable (*n*) is indicated in either figures, inside the bars, or figure legends. Parametric tests were used (performed using data obtained from pilot experiments) after confirming that the variables under analysis displayed Gaussian distributions using the D'Agostino-Pearson test (computed using GraphPad Prism 5). The significance is indicated as **** for *P*<0.0001, *** for *P*<0.001, ** for *P*<0.01 and * for *P*<0.05.

### Digital image processing

Fluorescence, transmission electron microscope and western blot images were acquired as uncompressed bitmapped digital data (TIFF format) and processed using Adobe Photoshop CS3 Extended, employing established scientific imaging workflows.^24^

## Abbreviations

BiP: binding immunoglobulin protein
eIF2*α*: eukaryotic translation initiation factor 2 alpha
ER: endoplasmic reticulum
dMfn: *Drosophila* mitofusin
PERK: protein kinase R-like endoplasmic reticulum kinase
PD: Parkinson's disease
PINK1: PTEN-induced putative kinase 1
PPL1: protocerebral posterior lateral 1
RNAi: RNA interference
UPR: unfolded protein response
Δψm: mitochondrial membrane potential.
